# Targeting Human Papillomavirus to Reduce the Burden of Cervical, Vulvar and Vaginal Cancer and Pre-Invasive Neoplasia: Establishing the Baseline for Surveillance

**DOI:** 10.1371/journal.pone.0088323

**Published:** 2014-02-05

**Authors:** Mari Nygård, Bo Terning Hansen, Joakim Dillner, Christian Munk, Kristján Oddsson, Laufey Tryggvadottir, Maria Hortlund, Kai-Li Liaw, Erik J. Dasbach, Susanne Krüger Kjær

**Affiliations:** 1 Department of Research, Cancer Registry of Norway, Oslo, Norway; 2 Departments of Laboratory Medicine, Medical Epidemiology and Biostatistics, Karolinska Institutet, Stockholm, Sweden; 3 Unit of Virus, Lifestyle and Genes, Danish Cancer Society Research Center, Copenhagen, Denmark; 4 The Cancer Detection Clinic, The Icelandic Cancer Society, Reykjavik, Iceland; 5 Icelandic Cancer Registry, Reykjavik, Iceland; 6 Faculty of Medicine, Laeknagardur, University of Iceland, Reykjavik, Iceland; 7 Office for Medical Service, Department of Clinical Microbiology, Division of Laboratory Medicine, Region Skåne, Malmö, Sweden; 8 Department of Epidemiology, Merck Research Laboratories, North Wales, Pennsylvania, United States of America; 9 Health Economic Statistics, Merck Research Laboratories, North Wales, Pennsylvania, United States of America; 10 Department of Gynecology, Rigshospitalet, University of Copenhagen, Copenhagen, Denmark; University of Nebraska Medical Center, United States of America

## Abstract

**Background:**

Infection with high-risk human papillomavirus (HPV) is causally related to cervical, vulvar and vaginal pre-invasive neoplasias and cancers. Highly effective vaccines against HPV types 16/18 have been available since 2006, and are currently used in many countries in combination with cervical cancer screening to control the burden of cervical cancer. We estimated the overall and age-specific incidence rate (IR) of cervical, vulvar and vaginal cancer and pre-invasive neoplasia in Denmark, Iceland, Norway and Sweden in 2004–2006, prior to the availability of HPV vaccines, in order to establish a baseline for surveillance. We also estimated the population attributable fraction to determine roughly the expected effect of HPV16/18 vaccination on the incidence of these diseases.

**Methods:**

Information on incident cervical, vulvar and vaginal cancers and high-grade pre-invasive neoplasias was obtained from high-quality national population-based registries. A literature review was conducted to define the fraction of these lesions attributable to HPV16/18, i.e., those that could be prevented by HPV vaccination.

**Results:**

Among the four countries, the age-standardised IR/10^5^ of cervical, vaginal and vulvar cancer ranged from 8.4–13.8, 1.3–3.1 and 0.2–0.6, respectively. The risk for cervical cancer was highest in women aged 30–39, while vulvar and vaginal cancers were most common in women aged 70+. Age-standardised IR/10^5^ of cervical, vulvar and vaginal pre-invasive neoplasia ranged between 138.8−183.2, 2.5−8.8 and 0.5−1.3, respectively. Women aged 20−29 had the highest risk for cervical pre-invasive neoplasia, while vulvar and vaginal pre-invasive neoplasia peaked in women aged 40−49 and 60−69, respectively. Over 50% of the observed 47,820 incident invasive and pre-invasive cancer cases in 2004−2006 can be attributed to HPV16/18.

**Conclusion:**

In the four countries, vaccination against HPV 16/18 could prevent approximately 8500 cases of gynecological cancer and pre-cancer annually. Population-based cancer and vaccination registries are essential to assess the predicted public health effects of HPV vaccination.

## Introduction

Persistent infection with high-risk human papillomavirus types (hrHPV) is a necessary cause of cervical cancer and pre-invasive neoplasia [1]. Hr HPV types have been detected in virtually all cervical cancers and cervical intraepithelial neoplasia grades 2 and 3 (CIN2/3) [2]–[4]; in 40–70% of all vulvar and vaginal cancers, and in about 85–90% of vulvar intraepithelial neoplasia grades 2 and 3 (VIN2/3) and vaginal intraepithelial neoplasia grades 2 and 3 (VaIN2/3) [5]; illustrating the fact that HPV is also causing a significant proportion of non-cervical anogenital neoplasias [6], [7].

The reported proportions of specific HPV types detected in cervical, vulvar and vaginal cancer and pre-invasive neoplasias vary widely. Suggested explanations for this variation include differences in the sensitivity of the HPV detection methods used [8], global disparity in HPV type distribution, as well as the difficulties of taking into account the strong effect of age on HPV positivity rates in studies that included different age groups [9], [10]. HPV16 is the most common type, and has been detected in 48–72% of cervical, 27–58% of vulvar and 46–77% of vaginal cancers. HPV18 has been detected in 11–22% of cervical, 2–10% of vulvar and 3–27% of vaginal cancers [2]–[4], [6], [7], [10]–[12]. HPV16 has been reported to be present in 49–81% of CIN2/3, VIN2/3 and VaIN2/3, whereas only 2–14% of these lesions test positive for HPV18.

Prophylactic vaccines against HPV16/18 have demonstrated high efficacy against the development of type-specific CIN2/3 and external genital lesions in various age groups [13]–[15]. HPV vaccines have been commercially available since 2006, and several countries have recently initiated HPV vaccination programmes for young girls to reduce the burden of HPV-related disease [16].

The population-based cancer registries in the Nordic countries have been sources for cancer statistics since the 1950s [17]. Cancer registration has been shown to be close to complete, timely and fairly accurate over time, and established routines for data quality assurance exist [18]. While the registration of cytological and histological diagnosis of cervical pre-invasive neoplasia has been of importance to audit cervical cancer screening programs, the public health demand for population-based data on pre-invasive neoplasias has been limited. In the present study, the overall and age-specific incidence of cervical, vulvar and vaginal cancer and CIN2/3, cervical adenocarcinoma *in situ* (AIS), VIN2/3 and VaIN2/3 in Denmark, Iceland, Norway and Sweden were estimated, prior to the availability of HPV vaccines, to establish a baseline for surveillance of the effect of mass HPV16/18 vaccination. The expected effect of HPV vaccination on the incidence of cervical, vulvar and vaginal cancer and pre-invasive neoplasia was estimated by calculation of HPV16/18 population attributable fractions.

## Materials and Methods

The data were analyzed anonymously and we used publicly available data from population-based registries. Denmark, Iceland, Norway and Sweden share an official policy which supports and funds mandatory cancer registration in population-based registries. The information available in these registries includes topography, morphology and date of diagnosis, as well as the personal identification number of the patient. Recorded data in all countries follow the International Classification of Diseases and Related Health Problems 10^th^ Revision (ICD-10) [19], [20].

### Definition of Incident Cervical, Vulvar and Vaginal Cancer and Pre-invasive Neoplasia

Women diagnosed with incident cervical vulvar and vaginal cancer and pre-invasive neoplasia between 1 January 2004 and 31 December 2006 were identified from the relevant national registries.

Only primary tumours of the cervix (ICD-10: C53), vulva (ICD-10: C51) and/or vagina (ICD-10: C52) were included. Incident cancer cases and dates of diagnoses were classified following international guidelines [18]. Histology codes 8010/2, 8070/2, 8076/2, 8077/2, 8140/2, 8140/1, 8140/2, 8560/2 [19], corresponding to CIN2/3, cervical adenocarcinoma *in situ*, VIN2/3, and VaIN2/3, as described in Tavassoli et al. [19], were used to identify women with incident pre-invasive neoplasia. For incident cervical intraepithelial neoplasia, the women had no history of histologically confirmed cervical abnormalities (CIN2/3, AIS, cervical cancer) for the past two calendar years. When a women had consecutive histological diagnoses of pre-invasive neoplasia at the same location, the most severe grade of pre-invasive neoplasia on the same location for a 2-year period was used. However, a diagnosis of incident pre-invasive neoplasia was disregarded among women who also had a diagnosis of cancer at the same location four months or less after the date of pre-invasive neoplasia. The same patient contributed multiple times as an incident case if a cancer or pre-invasive neoplasia was diagnosed at more than one anatomical site.

The population-based Pathology Data Bank was used to identify incident cases of cancer and pre-invasive neoplasia in Denmark. The Pathology Data Bank is a nationwide computerized register, containing information about all clinical cytological and histological examinations performed in the country [21]. In Iceland, the population-based database of the Cancer Detection Clinic, in which all histopathological results are registered, was used to identify women with pre-invasive neoplasia. Data for Icelandic, Norwegian and Swedish cancer cases were obtained from the respective national population-based cancer registries [18], [22]. To identify pre-invasive neoplasias in Norway, three sources were consulted: 1) the Histology Registry, which contains information on all morphological diagnoses from the cervix uteri since 2002; 2) the CIN Registry, which contains information on all treatment procedures for CIN performed in Norway since 1997; and 3) the Cancer Registry of Norway [23]. Data on pre-invasive neoplasia in Sweden were obtained from the Swedish National Cancer Registry, the National Screening Registry and the regional screening registry in Gothenburg [24]. In Sweden, VIN2 and VaIN2 is not reported to the registries, and therefore was not available for the present study. To identify the population at risk in each country, total female population figures as of 1 January in each year of interest were obtained from Statistics Denmark, Statistics Iceland, the National Population Register of Norway, and Statistics Sweden.

### Statistical Analyses

For Denmark, Iceland, Norway and Sweden the incidence rates (IR) of cervical, vulvar and vaginal cancer and pre-invasive neoplasia were estimated per 100,000 woman-years in 2004–2006, the period prior to availability of the HPV vaccine. The age-specific rate for five-year interval age groups *i*, denoted as r*_i_*, was obtained by dividing the number of events in each age group *d_i_* by the corresponding women-years of observation *Y_i_* and multiplying by 100.000:

*r_i_* = *d_i_/Y_i_* x 100.000.

Overall incidence rates were adjusted for age according to European Standard Population [25]. The estimates were presented by age groups: 0–19, 20–29, 30–39, 40–49, 50–59, 60–69 and 70 years and over, or age-standardised using the European standard population. The entire female population alive on 1 January of the year of interest was used as the number of women-years of observation. No censoring for incident cases was performed since the number of cases was relatively small and did not affect the estimates. Incidence rates during the period 2004–2006 were calculated cumulatively, i.e. as the total number of cases relative to the corresponding total number of woman-years of observation accumulated during 2004–2006.

#### The population attributable risk calculation

We estimated the total burden of cancerous gynaecological diseases which can be prevented by HPV16/18 vaccination, by multiplying the observed incidence of cervical, vulvar and vaginal cancers and pre-invasive neoplasias with the etiologic fraction, or population attributable fraction (PAF), an assumed fraction of the disease that would not have occurred had HPV16/18 been absent from the population. As the risk of cervical, vulvar, and vaginal cancer and pre-invasive neoplasia is high among HPV16/18-positive women, and as current knowledge suggests that the presence of HPV16/18 in these lesions is sufficient to infer causality [1], we defined the PAF as the proportion of cancers and pre-invasive neoplasias positive for HPV16/18, an approach which has also been used by others [26], [27]. We used recent review articles to define the proportion of cervical, vulvar and vaginal cancers and pre-invasive neoplasias positive for HPV16/18. To account for the highly variable prevalence point estimates, we provide a range of the lowest and highest estimate of HPV16/18 in cancer and pre-invasive neoplasia for PAF.

## Results

In 2004–2006, the country-specific age-standardised incidence rates (ASIR) of cervical cancer varied between 8.4/10^5^ and 13.8/10^5^, being 64% higher in Denmark (highest) compared to Sweden (lowest). The ASIR of cervical pre-invasive neoplasia, i.e. CIN2/3 and adenocarcinoma *in situ* combined, varied between 138.8 and 183.2, being 32% higher in Iceland (highest) compared to Norway (lowest). In all countries vulvar cancer and pre-invasive neoplasia were more common than vaginal cancer and pre-invasive vaginal neoplasia. ASIRs of vulvar and vaginal cancer were lowest in Iceland; vulvar cancer was highest in Denmark (3.1/10^5^) and vaginal cancer was highest in Sweden (0.6/10^5^). The ASIR of vulvar and vaginal pre-invasive neoplasia were lowest in Norway (4.8/10^5^ and 0.9/10^5^, respectively). The ASIR of vulvar pre-invasive neoplasia was almost twice as high in Iceland (8.8/10^5^), and 60% higher in Denmark (7.7/10^5^), compared to Norway (Table 1).

**Table 1:** Number of and age-adjusted (European population) incidence rates per 100,000 of cancer and pre-invasive neoplasia in cervix, vulva and vagina, for 2004–2006 in Denmark, Iceland, Norway and Sweden.

	Denmark		Iceland	Norway		Sweden	
Female population 2004–2006	8,203,231		443,352	7,015,877		13,690,608	
Number of cases 2004–2006							
Cervical cancer	1,234		43	889		1,304	
CIN2/3 and AIS^a^	12,888		864	9,397		18,218	
Vulvar cancer	360		6	279		414	
VIN2/3^b^	639		40	350		393*	
Vaginal cancer	58		1	50		133	
VaIN2/3^c^	111		5	69		75*	
Age-adjusted incidence rates per 100,000							
Cervical cancer	13.8		9.5	11.8		8.4	
CIN2/3 and AIS^a^	169.7		183.2	138.8		145.0	
Vulvar cancer	3.1		1.3	2.9		1.8	
VIN2/3^b^	7.7		8.8	4.8		2.5*	
Vaginal cancer	0.5		0.2	0.5		0.6	
VaIN2/3^c^	1.2		1.3	0.9		0.5*	

a CIN2/3 and AIS - cervical intraepithelial neoplasia grade 2 and 3 and adenocarcinoma *in situ*.

b VIN2/3 - vulvar intraepithelial neoplasia grade 2/3.

c VaIN2/3 - vaginal intraepithelial neoplasia grade 2/3.

*from Sweden only grade 3 VIN and VaIN were included.

**Figure 1:**
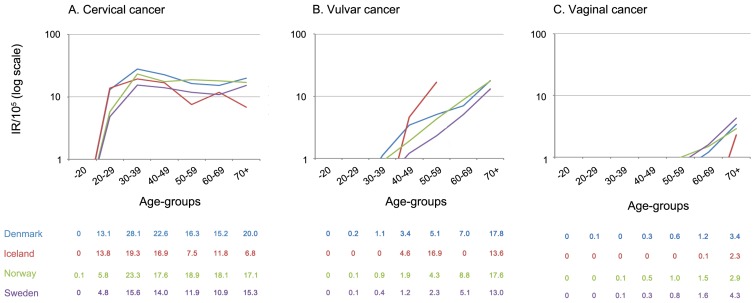
Incidence rates/10^5^ of cervical (A), vulvar (B) and vaginal (C) cancer in 2004–2006, by age and country. The y-axis shows incidence rates per 100,000 person years on a log scale and the x-axis represents age group. Blue, red, green, and violet lines refer to Denmark, Iceland, Norway and Sweden, respectively. Country specific point-estimates for incidence rate per 100,000 are given in a four-row table below each panel, by age-groups: 0–19, 20–29, 30–39, 40–49, 50–59, 60–69 and 70 years and over.

**Figure 2:**
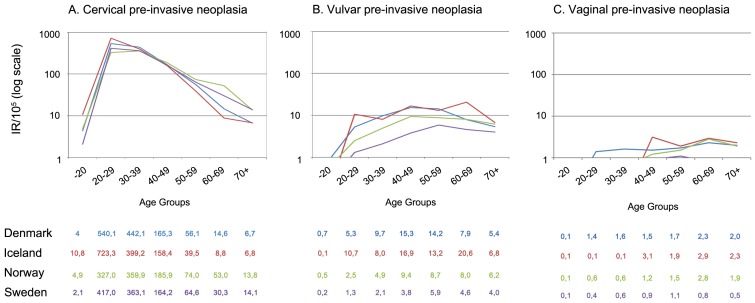
Incidence rates/10^5^ of cervical (A), vulvar (B) and vaginal (C) pre-invasive neoplasia in 2004–2006, by age and country. The y-axis shows incidence rates per 100,000 person years on a log scale and the x-axis represents age group. Blue, red, green, and violet lines refer to Denmark, Iceland, Norway and Sweden, respectively. Country specific point-estimates for incidence rate per 100,000 are given in a four-row table below each panel, sorted by age-groups: 0–19, 20–29, 30–39, 40–49, 50–59, 60–69 and 70 years and over. From Sweden only vulvar and vaginal intraepithelial neoplasia grade 3 are included, whereas other countries include both grades 2 and 3.

In all countries, the incidence of cervical cancer began to rise at age 20–29 years, and reached a peak at age 30–39 years, being highest in Denmark and lowest in Sweden (Figure 1A). The incidence of cervical pre-invasive neoplasia peaked earlier, at age 20–29 years, being highest in Iceland (723.3/10^5^) and lowest in Norway (327.0/10^5^) and declining markedly thereafter, to 10.4/10^5^ for women over 70 years of age, combined for all four countries (Figure 2A).

The incidence of vulvar and vaginal cancer started to increase at age 40–49 and 60–69 years, respectively, reaching a peak among women 70 years of age or older (Figure 1B and C). Vulvar pre-invasive neoplasia started to rise at age 20–29 years, reaching to a plateau at age 40 years or older (Figure 2B). Vaginal pre-invasive neoplasia was relatively uncommon in women younger than 50 years of age (Figure 2C), and in this age group incident vulvar and vaginal pre-invasive neoplasias were more common than cancers, whereas the opposite was true for women 70 years or older.

Altogether, 47,820 incident cases of cervical, vulvar and vaginal cancer and pre-invasive neoplasia were registered during the study period (2004–2006) in Denmark, Iceland, Norway and Sweden. Based on the published literature, the proportion of cases positive for HPV16/18 was defined and used as the PAF, which ranged between 71.2%–83.9% in cervical cancers, 30%–39% in vulvar cancers, 49.5%–100% in vaginal cancers; 51.5%–66.6% in cervical pre-invasive neoplasias, 64.5%–81.3% in vulvar pre-invasive neoplasia, 54.0%–88.6% in vaginal pre-invasive neoplasia (Table 2). During 2004–2006, between 2,909 and 3,566 incident cervical, vulvar and vaginal cancer cases, and between 22,361 and 28,936 cervical, vulvar and vaginal pre-invasive neoplasias were attributed to HPV16/18 in the four countries combined. Altogether, 32,226 women younger than 40 years and 15,594 women aged 40 years or older with incident pre-invasive or invasive lesions were registered. Among those, cases attributable to HPV16/18 ranged between 16,852 and 21,709 for women under 40 years of age and between 8,416 and 10,795 for women aged 40 years or older.

**Table 2:** Observed number of cervical, vulvar and vaginal cancer and pre-invasive neoplasia in,Denmark, Iceland, Norway and Sweden in 2004–2006, with estimated HPV16/18 population attributable fraction (PAF) and estimated absolute number of cases attributed to the HPV16/18.

Lesion type (reference)	HPV16/18 PAF %	−39 yrs of age		40+ yrs of age		All ages	
		Total no of cases	HPV16/18 attributable cases	Total noof cases	HPV16/18 attributable cases	Total noof cases	HPV16/18 attributable cases
Cervical cancer		1,114		2,356		3,470	
[2]–[4], [10], [11]	Lowest 71.2		793		1,677		2,471
	Highest 83.9		935		1,977		2,911
CIN2/3 and AIS^1^		30,683		10,684		41,367	
[2]–[4], [11]	Lowest 51.5		15,802		5,502		21,304
	Highest 66.6		20,435		7,116		27,550
Vulvar cancer		35		1,024		1,059	
[6], [7], [11], [12]	Lowest 30.0		11		307		318
	Highest 39.0		14		399		413
VIN2/3^2^		331		1,091		1,422	
[5]–[7], [11], [12]	Lowest 64.5		213		704		917
	Highest 81.3		269		887		1,156
Vaginal cancer		3		239		242	
[6], [7], [11], [12]	Lowest 49.5		1		118		120
	Highest 100.0		3		239		242
VaIN2/3^3^		60		200		260	
[6], [7], [11], [12]	Lowest 54.0		32		108		140
	Highest 88.6		53		177		230

1 CIN2/3 and AIS - cervical intraepithelial neoplasia grade 2 and 3 and adenocarcinoma *in situ*.

2 VIN2/3 - vulvar intraepithelial neoplasia grade 2/3; from Sweden only grade 3 VIN was included.

3 VaIN2/3 - vaginal intraepithelial neoplasia grade 2/3; from Sweden only grade 3 VaIN was included.

**Figure 3:**
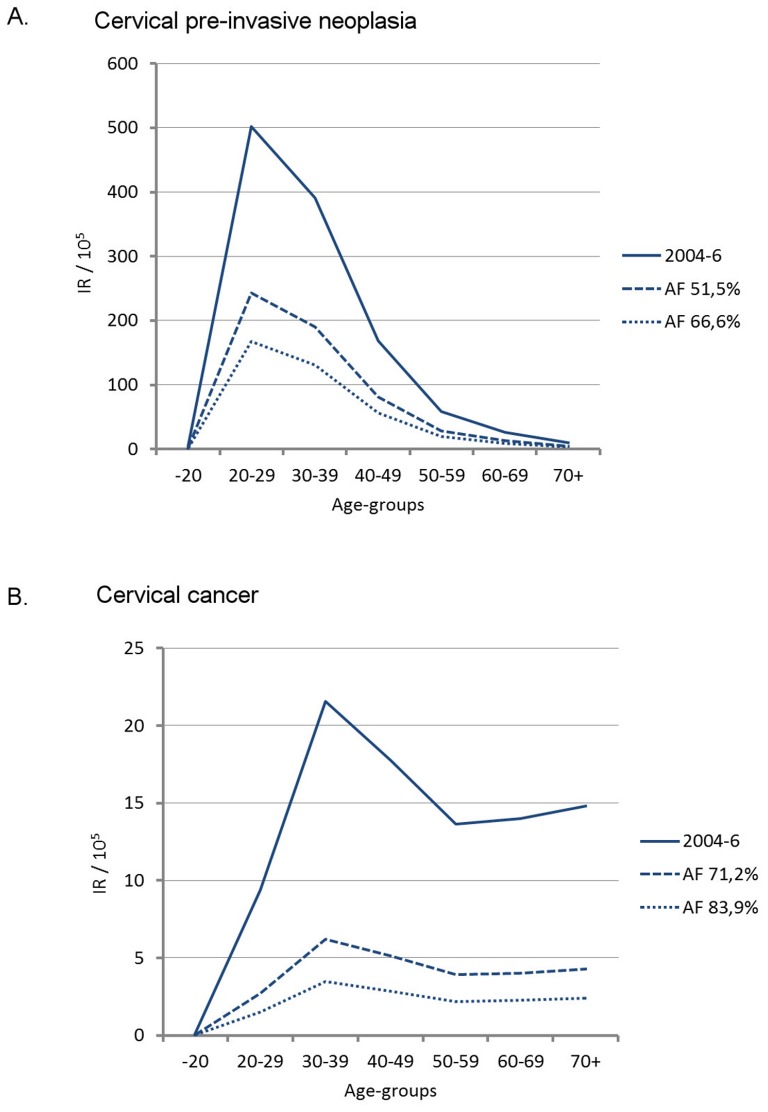
Observed and hypothetical incidence rate per 100,000 woman years of cervical pre-invasive nepolasia (A) and cervical cancer (B) by age in four Nordic countries combined in 2004–2006. The y-axis shows incidence rates per 100,000 person years and the x-axis represents age group. The solid line refers to observed incidence in 2004–2006; the long-dashed and short dashed line refers to expected incidence assuming the lowest and highest estimated HPV16/18 attributable fractions, respectively.

To illustrate the potential impact of HPV vaccination under the ideal assumption of 100% vaccine efficacy and coverage, the incidence of cervical cancer and pre-invasive neoplasia incidence were estimated (combined for all countries, by age). For women aged 20–29 years, where pre-invasive neoplasia was most frequent, the incidence of cervical pre-invasive neoplasia would be between 243.4/10^5^ and 167.6/10^5^ instead of the observed 501.9/10^5^, assuming the lowest and highest estimated HPV16/18 attributable fractions of 51.5% and 66.6%, respectively. For women aged 30–39 years, where cervical cancer is most frequent, the incidence of cervical cancer would be between 6.2/10^5^ and 3.5/10^5^ instead of the observed 21.6/10^5^, assuming the lowest and highest estimated HPV16/18 attributable fractions of 71.2% and 83.9%, respectively (Figure 3).

## Discussion

This study provides baseline incidence rates of HPV-related gynaecological lesions in four Nordic countries prior to the introduction of HPV vaccination. To our knowledge, this is the first time *empirical* population-based data on all HPV-related gynaecological cancer and pre-invasive neoplasia, including the cervix, vulva and vagina have been considered together for an estimation of the fraction potentially preventable by HPV16/18 vaccination.

While similar overall age-patterns of IRs of HPV-related gynaecological neoplasias in the four Nordic countries indicate the high quality of the national registry data, we observed that the ASIR of cervical pre-invasive neoplasia differed to a large extent between the countries. We have previously described sexual behaviour and smoking habits among women in these four Nordic countries [28]–[31], and the rather small differences described are unlikely to provide a full explanation for the variation in incidence of cervical pre-invasive neoplasia. Moreover, the prevalence of HPV-induced genital warts, was similar in Denmark, Iceland, Norway and Sweden [32]. National differences in the incidence of cervical intraepithelial neoplasia can also be explained by different cervical cancer screening recommendations. It has been shown that more intensive screening and follow-up leads to an increased detection of CIN2 or worse [33]. While all four countries have long-established national cervical cancer screening programs with high coverage, the target population and screening intensity differ between countries [34]. The lowest incidence of cervical pre-invasive neoplasia in the age-group of 20–29 years was found in Norway (327/10^5^), where screening starts at 25 years of age, and the highest was found in Iceland (723/10^5^), where screening starts at 20 years of age. Among age groups covered by the cervical cancer screening programmes in all four countries, the incidence of cervical pre-invasive neoplasia was comparable or showed only minor variation. Our study results, hence, imply that the registry data on cervical pre-invasive neoplasia does not necessarily reflect the true distribution of the disease in the population, but that it also depends on the intensity of routine screening applied in the population. Hence, the registered number of cervical pre-invasive neoplasia preventable by HPV vaccination is dependent on the existing screening activity in the respective country.

In the four countries combined, an annual average of about 1,160 and 14,000 women were diagnosed and treated for cervical cancer and pre-invasive neoplasia, respectively. A natural history study showed that about 30% of CIN3 lesions will progress to cancer after 30 years of follow-up if left untreated [35], indicating that about two-thirds of women with CIN3 might be over-treated. The rate of overtreatment may be even higher in our population, assuming that a proportion of the pre-invasive lesions were CIN2, and that CIN2 has a lower cancer progression risk than CIN3. Although generally well tolerated, treatment for cervical pre-invasive neoplasia increases the risk of preterm delivery, which can adversely affect the health of the new-born [36], [37]. Clearly, these considerations are highly relevant to women of reproductive age. Hence, in countries with organized cervical screening programs HPV vaccination has a great potential for reducing over-treatment of CIN2/3.

An effective HPV vaccination programme would decrease the incidence of cervical pre-invasive neoplasia by 51.5% to 66.6%, based on the literature review. This is in line with a recently published study suggesting a reduction of CIN2/3 by 58.6% to 62.1%, where the estimates were derived from a comprehensive mathematical micro-simulation model integrating complexity across the HPV-natural history model as well as HPV-related disease control, such as screening activity and HPV vaccination coverage [38]. Under ideal circumstances, i.e. 100% immunisation of the population at risk, pre-invasive cervical neoplasia would be reduced from 502/10^5^ to 168–243/10^5^ among women 20–29 years old, and from 391/10^5^ to 131–190/10^5^ among women 30–39 years old. In absolute terms, this translates into a total of 21,304–27,550 fewer women treated over 3 years, implying a reduced demand for diagnostic and treatment facilities, and improved reproductive health. The goal of HPV vaccination is to reduce the number of cervical cancer cases, which can be achieved for 71–84% of the cases according to our estimate. This translates into about 2,471 to 2,911 fewer cervical cancer cases to be diagnosed and treated triennially in the four Nordic countries combined.

However, the effect of HPV vaccination programmes on cancer incidence rates will not be observable at the population level until the birth cohorts that have received the vaccine prior to HPV exposure/sexual activity reach their thirties. Different countries have different policies for HPV vaccination, which causes variation in vaccine coverage across birth cohorts [39]. According to a published mathematical model it may take as long as 40 years after the initiation of HPV vaccination programmes for reductions in cancer incidence rates to be observed [38]. However, the effect of HPV vaccination programmes on the incidence of cervical pre-invasive neoplasia, as well as on procedures related to screening, diagnostics and therapy, will be observable decades sooner. Concurrent improvement of screening programmes through the use of HPV testing in primary screening or in triage might, however, have the opposite effect, resulting in an increase of the overall occurrence of cervical pre-invasive neoplasia. Randomised studies showed that CIN2 or worse was detected 30–80% more at baseline and 47–57% less at the 2^nd^ screening round in the HPV arm as compared to the cytology arm [40]–[42]. Furthermore, it is highly likely that after a negative HPV screening test, the screening interval will be extended beyond what was used in the controlled studies, and incorporation of self-sampling may improve the coverage and performance of screening programmes [43]–[45]. Mathematical models evaluating the cost-effectiveness of alternative screening and HPV vaccination strategies also favour a switch to primary HPV testing among women over 30 years of age [46]. Therefore, it is plausible that HPV-based screening will replace cytology screening in the near future, which inevitably will introduce changes in the epidemiology of cervical cancer and pre-invasive neoplasia. The interpretation of trends in the IRs of cervical pre-invasive neoplasia at the population level can be extremely difficult without information on HPV vaccination. Meticulous registration covering all aspects of cervical cancer prevention is therefore highly recommended to correctly interpret the forthcoming changes in the epidemiology of cervical pre-invasive neoplasia.

Compared to cervical cancer, vulvar and vaginal cancer are rare diseases, commonly affecting women older than 70 years of age. During 2004–2006 more than 1,050 vulvar and 240 vaginal cancer cases were diagnosed in the four Nordic countries combined, which corresponds to about a third of the cervical cancer cases observed. Since vulvar and vaginal cancers tend to occur among older women, and the HPV16/18 attributable fraction is low for vulvar cancer (30–39%), the impact of HPV vaccination will not be observable for many decades. Vulvar pre-invasive neoplasia was most common among women 40–49 years of age, with an IR of 11.4/10^5^. This is in line with studies reporting a mean age of 48 years at HPV-related diagnosis of vulvar pre-invasive neoplasia [47], [48]. However, in our study, much higher rates of vulvar pre-invasive neoplasia were reported compared to the Netherlands (2/10^5^) [47]. It remains unclear to what extent the observed discrepancies can be explained by the completeness of registries, differences in clinical practice and management [23], or different background risk. Although HPV16/18 PAF estimates for vulvar and vaginal pre-invasive neoplasia were comparable to those in the cervix, the major public health advantage of HPV16/18 vaccination lies in the prevention of cervical lesions since vulvar and vaginal lesions affect far fewer women. Moreover, pre-invasive and invasive lesions of the vulva and vagina tend to affect much older women than do the corresponding cervical lesions. Nevertheless, the personal gain of HPV vaccination on an individual level cannot be ignored, as there is currently no effective treatment available for vulvar pre-invasive lesions [49].

The ultimate aim of HPV vaccination is to reduce the burden of all HPV-related diseases. HPV16 also seems to be causally associated with anal, penile and oro-pharyngeal neoplasias [50]–[53], thus HPV16/18 vaccination may also reduce the incidence of these diseases. The quadrivalent HPV vaccine additionally protects against HPV6/11, which cause genital warts [13] and recurrent papillomatosis [54]. Empirical observations form from Australia, Denmark and Sweden, where comprehensive population-based HPV vaccination programmes were introduced some years ago, have already demonstrated changes in the epidemiology of genital warts at the population level [55]–[57].

In order to monitor and evaluate the effect of nation-wide HPV vaccination programs, it is necessary to define realistic targets, such as the expected decrease in the burden of HPV-related gynaecological cancers relative to a baseline incidence. During the transition period, in which partially vaccinated birth cohorts enter the screening programs, changes in population-based incidence rates of cervical pre-invasive neoplasia can be difficult to interpret because the incidence of CIN2 or worse will reflect an interplay between underlying risk factors, screening and HPV vaccination. Therefore, it is important to establish integrated population-based registration of all aspects of cervical cancer screening and HPV vaccination activities, and to continuously evaluate changes in the epidemiology of HPV-related diseases.

## References

[1] IARC (2007) Human Papillomaviruses. Lyon: WHO Press. 978–92–832–1290–4 978–92–832–1290–4.

[2] De Vuyst H, Clifford G, Li N, Franceschi S (2009) HPV infection in Europe. Eur J Cancer 45: 2632–2639. 19709878 10.1016/j.ejca.2009.07.019

[3] Smith JS, Lindsay L, Hoots B, Keys J, Franceschi S, et al (2007) Human papillomavirus type distribution in invasive cervical cancer and high-grade cervical lesions: a meta-analysis update. Int J Cancer 121: 621–632. 17405118 10.1002/ijc.22527

[4] Kjaer SK, Breugelmans G, Munk C, Junge J, Watson M, et al (2008) Population-based prevalence, type- and age-specific distribution of HPV in women before introduction of an HPV-vaccination program in Denmark. Int J Cancer 123: 1864–1870. 18661520 10.1002/ijc.23712

[5] van de Nieuwenhof HP, van der Avoort IA, de Hullu JA (2008) Review of squamous premalignant vulvar lesions. Crit Rev Oncol Hematol 68: 131–156. 18406622 10.1016/j.critrevonc.2008.02.012

[6] De Vuyst H, Clifford GM, Nascimento MC, Madeleine MM, Franceschi S (2009) Prevalence and type distribution of human papillomavirus in carcinoma and intraepithelial neoplasia of the vulva, vagina and anus: a meta-analysis. Int J Cancer 124: 1626–1636. 19115209 10.1002/ijc.24116

[7] Garland SM, Insinga RP, Sings HL, Haupt RM, Joura EA (2009) Human papillomavirus infections and vulvar disease development. Cancer Epidemiol Biomarkers Prev 18: 1777–1784. 19505910 10.1158/1055-9965.EPI-09-0067

[8] Chao FY, Chao A, Huang CC, Hsueh S, Yang JE, et al (2010) Defining detection threshold and improving analytical proficiency of HPV testing in clinical specimens. Gynecol Oncol 117: 302–307. 20206982 10.1016/j.ygyno.2010.02.001

[9] de Sanjose S, Diaz M, Castellsague X, Clifford G, Bruni L, et al (2007) Worldwide prevalence and genotype distribution of cervical human papillomavirus DNA in women with normal cytology: a meta-analysis. Lancet Infect Dis 7: 453–459. 17597569 10.1016/S1473-3099(07)70158-5

[10] de Sanjose S, Quint WG, Alemany L, Geraets DT, Klaustermeier JE, et al (2010) Human papillomavirus genotype attribution in invasive cervical cancer: a retrospective cross-sectional worldwide study. Lancet Oncol 11: 1048–1056. 20952254 10.1016/S1470-2045(10)70230-8

[11] Insinga RP, Liaw KL, Johnson LG, Madeleine MM (2008) A systematic review of the prevalence and attribution of human papillomavirus types among cervical, vaginal, and vulvar precancers and cancers in the United States. Cancer Epidemiol Biomarkers Prev 17: 1611–1622. 18628412 10.1158/1055-9965.EPI-07-2922 PMC2587113

[12] Smith JS, Backes DM, Hoots BE, Kurman RJ, Pimenta JM (2009) Human Papillomavirus Type-Distribution in Vulvar and Vaginal Cancers and Their Associated Precursors. Obstet Gynecol 113: 917–924. 19305339 10.1097/AOG.0b013e31819bd6e0

[13] Ault KA (2007) Effect of prophylactic human papillomavirus L1 virus-like-particle vaccine on risk of cervical intraepithelial neoplasia grade 2, grade 3, and adenocarcinoma in situ: a combined analysis of four randomised clinical trials. Lancet 369: 1861–1868. 17544766 10.1016/S0140-6736(07)60852-6

[14] Munoz N, Manalastas R Jr, Pitisuttithum P, Tresukosol D, Monsonego J, et al (2009) Safety, immunogenicity, and efficacy of quadrivalent human papillomavirus (types 6, 11, 16, 18) recombinant vaccine in women aged 24–45 years: a randomised, double-blind trial. Lancet 373: 1949–1957. 19493565 10.1016/S0140-6736(09)60691-7

[15] Paavonen J, Naud P, Salmeron J, Wheeler CM, Chow SN, et al (2009) Efficacy of human papillomavirus (HPV)-16/18 AS04-adjuvanted vaccine against cervical infection and precancer caused by oncogenic HPV types (PATRICIA): final analysis of a double-blind, randomised study in young women. Lancet 374: 301–314. 19586656 10.1016/S0140-6736(09)61248-4

[16] Brotherton J, Gertig D, Chappell G, Rowlands L, Saville M (2011) Catching up with the catch-up: HPV vaccination coverage data for Australian women aged 18–26 years from the National HPV Vaccination Program Register. Commun Dis Intell 35: 197–201. 10.33321/cdi.2011.35.18 22010515

[17] Moller B, Fekjaer H, Hakulinen T, Tryggvadottir L, Storm HH, et al (2002) Prediction of cancer incidence in the Nordic countries up to the year 2020. Eur J Cancer Prev 11 Suppl 1S1–96. 12442806

[18] Larsen IK, Smastuen M, Johannesen TB, Langmark F, Parkin DM, et al (2009) Data quality at the Cancer Registry of Norway: an overview of comparability, completeness, validity and timeliness. Eur J Cancer 45: 1218–1231. 19091545 10.1016/j.ejca.2008.10.037

[19] Tavassoli F, Devilee P, editors (2003) Pathology and Genetics of Tumours of the Breast and Female Genital Organs. Lyon: IARC*Press*.

[20] WHO (1992) The ICD-10 classification of mental and behavioural disorders. Clinical descriptions and diagnostic guidelines. Geneva: World Health Organization. 362 p.

[21] Bjerregaard B, Larsen OB (2011) The Danish Pathology Register. Scand J Public Health 39: 72–74. 21775357 10.1177/1403494810393563

[22] Sigurdsson K (2010) Cervical cancer: cytological cervical screening in Iceland and implications of HPV vaccines. Cytopathology 21: 213–222. 20646020 10.1111/j.1365-2303.2010.00783.x

[23] Enerly E, Bray F, Mellem C, Hansen BT, Kjolberg G, et al (2012) Quality assessment of the registration of vulvar and vaginal premalignant lesions at the Cancer Registry of Norway. Acta Oncol 51: 45–50. 22047060 10.3109/0284186X.2011.624545 PMC3251004

[24] Andrae B, Kemetli L, Sparen P, Silfverdal L, Strander B, et al (2008) Screening-preventable cervical cancer risks: evidence from a nationwide audit in Sweden. J Natl Cancer Inst 100: 622–629. 18445828 10.1093/jnci/djn099

[25] dos Santos Silva I (1999) Cancer Epidemiology: Principles and Methods. Lyon: IARC.

[26] de Martel C, Ferlay J, Franceschi S, Vignat J, Bray F, et al (2012) Global burden of cancers attributable to infections in 2008: a review and synthetic analysis. Lancet Oncol 13: 607–615. 22575588 10.1016/S1470-2045(12)70137-7

[27] Miettinen OS (1974) Proportion of disease caused or prevented by a given exposure, trait or intervention. Am J Epidemiol 99: 325–332. 4825599 10.1093/oxfordjournals.aje.a121617

[28] Eliasen M, Kaer SK, Munk C, Nygard M, Sparen P, et al (2009) The relationship between age at drinking onset and subsequent binge drinking among women. Eur J Public Health 19: 378–382. 19321715 10.1093/eurpub/ckp023

[29] Hansen BT, Hagerup-Jenssen M, Kjaer SK, Munk C, Tryggvadottir L, et al (2010) Association between smoking and genital warts: longitudinal analysis. Sex Transm Infect 86: 258–262. 20660589 10.1136/sti.2009.038273

[30] Jensen KE, Munk C, Sparen P, Tryggvadottir L, Liaw KL, et al (2011) Women’s sexual behavior. Population-based study among 65,000 women from four Nordic countries before introduction of human papillomavirus vaccination. Acta Obstet Gynecol Scand 90: 459–467. 21306319 10.1111/j.1600-0412.2010.01066.x

[31] Olesen TB, Jensen KE, Nygard M, Tryggvadottir L, Sparen P, et al (2012) Young age at first intercourse and risk-taking behaviours–a study of nearly 65 000 women in four Nordic countries. Eur J Public Health 22: 220–224. 21596800 10.1093/eurpub/ckr055

[32] Kjaer SK, Tran TN, Sparen P, Tryggvadottir L, Munk C, et al (2007) The burden of genital warts: a study of nearly 70,000 women from the general female population in the 4 Nordic countries. J Infect Dis 196: 1447–1454. 18008222 10.1086/522863

[33] Nygard JF, Nygard M, Skare GB, Thoresen SO (2006) Pap smear screening in women under 30 in the Norwegian Coordinated Cervical Cancer Screening Program, with a comparison of immediate biopsy vs Pap smear triage of moderate dysplasia. Acta Cytol 50: 295–302. 16780024 10.1159/000325957

[34] Sigurdsson K (1999) The Icelandic and Nordic cervical screening programs: trends in incidence and mortality rates through 1995. Acta Obstetricia et Gynecologica Scandinavica 78: 478–485. 10376856

[35] McCredie MR, Sharples KJ, Paul C, Baranyai J, Medley G, et al (2008) Natural history of cervical neoplasia and risk of invasive cancer in women with cervical intraepithelial neoplasia 3: a retrospective cohort study. Lancet Oncol 9: 425–434. 18407790 10.1016/S1470-2045(08)70103-7

[36] Albrechtsen S, Rasmussen S, Thoresen S, Irgens LM, Iversen OE (2008) Pregnancy outcome in women before and after cervical conisation: population based cohort study. BMJ 337: a1343. 18801869 10.1136/bmj.a1343 PMC2544429

[37] Nohr B, Tabor A, Frederiksen K, Kjaer SK (2007) Loop electrosurgical excision of the cervix and the subsequent risk of preterm delivery. Acta Obstet Gynecol Scand 86: 596–603. 17464590 10.1080/00016340701279145

[38] Van de Velde N, Boily MC, Drolet M, Franco EL, Mayrand MH, et al (2012) Population-level impact of the bivalent, quadrivalent, and nonavalent human papillomavirus vaccines: a model-based analysis. J Natl Cancer Inst 104: 1712–1723. 23104323 10.1093/jnci/djs395

[39] Levy-Bruhl D, Bousquet V, King LA, O’Flanagan D, Bacci S, et al (2009) The current state of introduction of HPV vaccination into national immunisation schedules in Europe: results of the VENICE 2008 survey. Eur J Cancer 45: 2709–2713. 19695863 10.1016/j.ejca.2009.07.023

[40] Bulkmans NW, Berkhof J, Rozendaal L, van Kemenade FJ, Boeke AJ, et al (2007) Human papillomavirus DNA testing for the detection of cervical intraepithelial neoplasia grade 3 and cancer: 5-year follow-up of a randomised controlled implementation trial. Lancet 370: 1764–1772. 17919718 10.1016/S0140-6736(07)61450-0

[41] Naucler P, Ryd W, Tornberg S, Strand A, Wadell G, et al (2007) Human papillomavirus and Papanicolaou tests to screen for cervical cancer. N Engl J Med 357: 1589–1597. 17942872 10.1056/NEJMoa073204

[42] Ronco G, Giorgi-Rossi P, Carozzi F, Confortini M, Dalla Palma P, et al (2010) Efficacy of human papillomavirus testing for the detection of invasive cervical cancers and cervical intraepithelial neoplasia: a randomised controlled trial. Lancet Oncol 11: 249–257. 20089449 10.1016/S1470-2045(09)70360-2

[43] Kjaer SK, Frederiksen K, Munk C, Iftner T (2010) Long-term absolute risk of cervical intraepithelial neoplasia grade 3 or worse following human papillomavirus infection: role of persistence. J Natl Cancer Inst 102: 1478–1488. 20841605 10.1093/jnci/djq356 PMC2950170

[44] Saslow D, Solomon D, Lawson HW, Killackey M, Kulasingam SL, et al (2012) American Cancer Society, American Society for Colposcopy and Cervical Pathology, and American Society for Clinical Pathology screening guidelines for the prevention and early detection of cervical cancer. CA Cancer J Clin 62: 147–172. 22422631 10.3322/caac.21139 PMC3801360

[45] Dijkstra MG, Heideman DA, van Kemenade FJ, Hogewoning KJ, Hesselink AT, et al (2012) Brush-based self-sampling in combination with GP5+/6+-PCR-based hrHPV testing: High concordance with physician-taken cervical scrapes for HPV genotyping and detection of high-grade CIN. J Clin Virol 54: 147–151. 22445557 10.1016/j.jcv.2012.02.022

[46] de Blasio BF, Neilson AR, Klemp M, Skjeldestad FE (2012) Modeling the impact of screening policy and screening compliance on incidence and mortality of cervical cancer in the post-HPV vaccination era. J Public Health (Oxf) 34: 539–547. 22707556 10.1093/pubmed/fds040

[47] van de Nieuwenhof HP, Massuger LF, van der Avoort IA, Bekkers RL, Casparie M, et al (2009) Vulvar squamous cell carcinoma development after diagnosis of VIN increases with age. Eur J Cancer 45: 851–856. 19117749 10.1016/j.ejca.2008.11.037

[48] Joura EA (2002) Epidemiology, diagnosis and treatment of vulvar intraepithelial neoplasia. Curr Opin Obstet Gynecol 14: 39–43. 11801875 10.1097/00001703-200202000-00007

[49] Kenter GG, Welters MJ, Valentijn AR, Lowik MJ, Berends-van der Meer DM, et al (2009) Vaccination against HPV-16 Oncoproteins for Vulvar Intraepithelial Neoplasia. N Engl J Med 361: 1838–1847. 19890126 10.1056/NEJMoa0810097

[50] Moscicki AB, Ma Y, Farhat S, Jay J, Hanson E, et al (2013) 27. Natural history of anal HPV in heterosexual women and risks associated with persistence. Sex Health 10: 583. 10.1093/cid/cit947 PMC3935503 24368624

[51] Ouhoummane N, Steben M, Coutlee F, Vuong T, Forest P, et al.. (2013) Squamous anal cancer: Patient characteristics and HPV type distribution. Cancer Epidemiol. 10.1016/j.canep.2013.09.015 24139594

[52] Scudellari M (2013) HPV: Sex, cancer and a virus. Nature 503: 330–332. 24256791 10.1038/503330a

[53] Mannweiler S, Sygulla S, Winter E, Regauer S (2013) Two major pathways of penile carcinogenesis: HPV-induced penile cancers overexpress p16ink4a, HPV-negative cancers associated with dermatoses express p53, but lack p16ink4a overexpression. J Am Acad Dermatol 69: 73–81. 23474228 10.1016/j.jaad.2012.12.973

[54] Chirila M, Bolboaca SD (2013) Clinical efficiency of quadrivalent HPV (types 6/11/16/18) vaccine in patients with recurrent respiratory papillomatosis. Eur Arch Otorhinolaryngol. 10.1007/s00405-013-2755-y 24121781

[55] Read TR, Hocking JS, Chen MY, Donovan B, Bradshaw CS, et al (2011) The near disappearance of genital warts in young women 4 years after commencing a national human papillomavirus (HPV) vaccination programme. Sex Transm Infect 87: 544–547. 21970896 10.1136/sextrans-2011-050234

[56] Baandrup L, Blomberg M, Dehlendorff C, Sand C, Andersen KK, et al (2013) Significant decrease in the incidence of genital warts in young danish women after implementation of a national human papillomavirus vaccination program. Sex Transm Dis 40: 130–135. 23324976 10.1097/OLQ.0b013e31827bd66b

[57] Leval A, Herweijer E, Ploner A, Eloranta S, Fridman Simard J, et al.. (2013) Quadrivalent Human Papillomavirus Vaccine Effectiveness: A Swedish National Cohort Study. J Natl Cancer Inst. 10.1093/jnci/djt032 PMC3614506 23486550

